# Genetics and genomics in Peru: Clinical and research perspective

**DOI:** 10.1002/mgg3.533

**Published:** 2018-12-25

**Authors:** Heinner Guio, Julio A. Poterico, Kelly S. Levano, Mario Cornejo‐Olivas, Pilar Mazzetti, Gioconda Manassero‐Morales, Manuel F. Ugarte‐Gil, Eduardo Acevedo‐Vásquez, Milagros Dueñas‐Roque, Alejandro Piscoya, Ricardo Fujita, Cesar Sanchez, Sandro Casavilca‐Zambrano, Luis Jaramillo‐Valverde, Yasser Sullcahuaman‐Allende, Juan M. Iglesias‐Pedraz, Hugo Abarca‐Barriga

**Affiliations:** ^1^ Instituto Nacional de Salud del Perú Lima Perú; ^2^ Universidad Científica del Sur Lima Perú; ^3^ Servicio de Genética Instituto Nacional de Salud del Niño San Borja (INSN‐SB) Lima Peru; ^4^ INBIOMEDIC Research and Technological Center Lima Peru; ^5^ Neurogenetics Research Center, Instituto Nacional de Ciencias Neurológicas Lima Perú; ^6^ School of Medicine Universidad Nacional Mayor de San Marcos Lima Perú; ^7^ Rheumatology Department. Hospital Guillermo Almenara Irigoyen. EsSalud Lima Perú; ^8^ Clínica San Felipe Lima Perú; ^9^ Servicio de Genética Hospital Nacional Edgardo Rebagliati Martins Lima Perú; ^10^ Sociedad de Genética Médica del Perú Lima Peru; ^11^ Universidad San Ignacio de Loyola Lima Perú; ^12^ Hospital Guillermo Kaelin de la Fuente Lima Perú; ^13^ Centro de Genética y Biología Molecular, Universidad de San Martín de Porres Lima Perú; ^14^ Banco de Tejidos Tumorales, Instituto Nacional de Enfermedades Neoplásicas Banco de Tejidos Tumorales Lima Perú; ^15^ Instituto Nacional de Enfermedades Neoplásicas Lima Perú; ^16^ Universidad Peruana de Ciencias Aplicadas UPC Lima Peru; ^17^ Laboratorio de Genética Molecular y Bioquímica, Departamento de Investigación, Desarrollo e Innovación Universidad Científica del Sur Lima Perú; ^18^ Servicio de Genética & EIM Instituto Nacional de Salud del Niño Breña (INSN) Lima Peru; ^19^ Facultad de Estomatología Universidad Científica del Sur Lima Perú; ^20^ Facultad de Medicina Humana Universidad Ricardo Palma Lima Perú

**Keywords:** genetic, genomic, Peru

## Abstract

Peruvians currently preserve in their DNA the history of 2.5 million years of human evolution and 150,000 years of migration from Africa to Peru or the Americas. The development of Genetics and Genomics in the clinical and academic field is shown in this review.

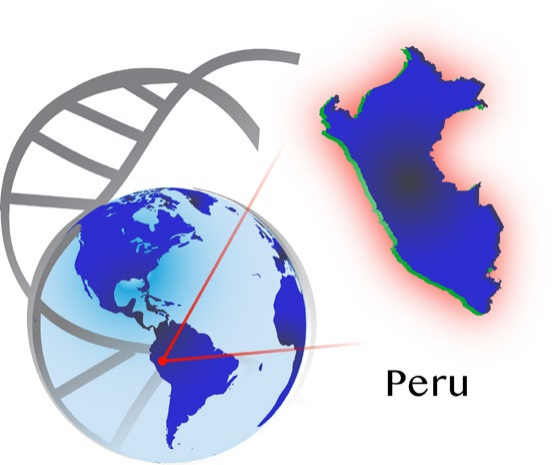

## 1 PERUVIAN DEMOGRAPHICS

Peru is a pluricultural and ethnically diverse country located in middle west of South America. It is the fourth largest country (1,285,216 km^2^) in area in South America, bordered in the north by Ecuador and Colombia, in the south by Chile, south east by Bolivia, by Brazil in the east and by the Pacific Ocean in the west. Peru has one of the most diverse climates, geographical and ecological in the world, from tropical or subtropical dessert to glacial in highland mountains to Amazon jungle. Peru is divided into 24 geopolitical regions with a central government. According to last national census in 2017, Peruvian populations accounts for 31,237,385 inhabitants (Instituto Nacional de Estadistica e Informatica [INEI], 2017). Most of Peru's population lives in the Coastal area, with almost one third of the nation's population living in Lima, the largest city with approximately 11 million people. Rural and urban population has changed over the last 50 years due to migration waves in the 1950s from rural to urban sites mainly located in the coast causing decrease or disappearance of native communities.

The Peruvian health care system in Peru includes five different subsystems: (a) Ministry of Health that provides services to people categorized as poor and extremely poor (around of the population) covered by SIS (Integral Health insurance), (b) Social security subsystem (EsSalud) administered by Labor and Employment Minister that provides services to formal employees and their dependents (26.7% of population), (c) Armed Forces subsystem run by Minister of Defense, (d) Police forces subsystem administered by Ministry of Interior and (e) the Private sector (Alcalde‐Rabanal, Lazo‐González, & Nigenda, 2011; Ypanaqué‐Luyo & Martins, 2015). Within the most prevalent diseases in Peru, upper respiratory infectious (viral or bacterial) diseases have the highest morbidity rates and cancer (gastric, cervical, uterine, breast, prostate, and lung) the highest mortality rates (INEI, 2015). This gives the unique opportunity for diverse genetic studies in the most prevalent infectious and non‐infectious diseases afflicting the Peruvian population.

## 2 HEALTHCARE SERVICES OF GENETICS AND GENOMICS

Genetic services in Peru are scarce but growing despite limitations in infrastructure and qualified human resources. The very few public genetic services available in the country offer clinical care and genetic counseling provided by medical geneticists and other specialists with training on genetics. Available molecular diagnosis is mostly based on cytogenetic techniques, PCR‐based and Sanger sequencing technologies. There are 21 accredited medical geneticists in national registry of the Peruvian Medical college of Physician (https://cmp.org.pe/conoce-a-tu-medico/) most of them working in public hospitals within the Capital city. Training in human genetics and medical genetics are scarce with only one accredited medical genetics residency program for physicians, one postgraduate program on human genetics and one biology, genetics and biotechnology graduate program for molecular biology specialists. Genetic counseling is currently offered by medical geneticists and other specialists with training in genetics; however, there is no formal accredited Genetic counseling program in Peru. In addition, national and international collaborations have been crucial specially in bioinformatic analysis due to in Peru there is still no university bioinformatics career, the trainings only are part of courses, seminars inside and outside our country.

### 2.1 Newborn screening

There are isolated hospital initiatives for newborn screening in Peru. By 2012, a law state the national interest for having a Universal Newborn screening program, prioritizing screening for congenital hypothyroidism (CH), congenital adrenal hyperplasia (CAH), phenylketonuria (PKU), cystic fibrosis, hearing loss and congenital cataract (Galán‐Rodas, Duenas, Obando, & Saborio 2013). Up to now, there are independent newborn screening programs in main hospitals of Ministry of Health, Social security (EsSalud) subsystem and private sector. The program from EsSAlud started back in 2011 (INEI, 2015), including screening for CH, PKU, CAH, and galactosemia (GAL) available in every single EsSalud healthcare center across the country. The incidence of CH, CAH, PKU, and GAL was 1:3,211, 1:22,207, 1:46,970, and 1:37,576, respectively (Data according to Dr. M. Dueñas). However, Peru has not yet completely implemented National Newborn Screening Program in Public Medical Centers.

### 2.2 Surveillance of birth defects

Since 2012, there is a hospital‐based registry for birth defects at the tertiary hospital of the social security subsystem, which is part of contributing to the Latin American Collaborative Study of Congenital Malformations (ECLAMC) (Cruz‐Ticona et al., 2015), The most common birth defects are as follows: congenital heart defects, Down syndrome, cleft lip and/or palate, hydrocephaly and neural tube defects.

### 2.3 Pediatric genetics

Clinical genetics started at the Hospital Guillermo Almenara Irigoyen in 1968, with clinical dysmorphology and karyotype diagnostic test without banding (Delgado‐Matallana, 2000). Furthermore, clinical dysmorphology flourished at Instituto Nacional de Salud del Niño‐Breña (INSN‐Breña), with the advantage of being the representative national institute for children's health. Over the years, all this experience was published as a unique book in dysmorphology linking patient (authorized by parents) images and a succinct clinical and molecular explanation of diseases. This recompilation entitled “Atlas de Dismorfología Pediátrica” (Atlas of Pediatric Dysmorphology) gathered more than 200 syndromes and recognizable clinical cases with Peruvian patients’ pictures (Klein, Gallardo, Chavez, & Abarca, 2012). Furthermore, INSN‐Breña is one of the biggest national institutions where patients from Peru and within the MINSA healthcare system receive diagnosis and management. In Table 1, we can observe the frequency of diagnosed genetic disorders in this institution at the Service of Genetic and Inborn Error of Metabolism.

**Table 1:** Frequency of genetic disorders diagnosed in the Peruvian National Institute of Health Breña (INSN‐Breña) from 2014 to 2018

Genetic disorders	Diagnostic counts^a^	Percentage
Down syndrome	1,344	12.69
Psychomotor retardation	582	5.49
Intellectual disability	520	4.91
Syndromic psychomotor delay	342	3.23
Syndrome Ehlers‐Danlos hypermobile	280	2.64
Syndromic intellectual disability	267	2.52
Autism spectrum disorder	251	2.37
Short size	202	1.91
Syndromic short	199	1.88
Language delay	191	1.8
Neurofibromatosis 1	159	1.5
Syndromic microcephaly	150	1.42
Microtia	142	1.34
Turner syndrome	128	1.21
Hemihyperplasia	117	1.1
Cleft lip‐palate	116	1.09
Malformation syndrome	112	1.06
Goldenhar syndrome	107	1.01
Macules café‐au‐lait	106	1
Skeletal dysplasia	101	0.95
Noonan syndrome	96	0.91
Imperfect osteogenesis	82	0.77
Microcephaly	75	0.71
Moebius syndrome	72	0.68
Hypotonia	58	0.55
Neural tube defect	56	0.53
Epilepsy	56	0.53
Marfanoid syndrome	56	0.53
Disorder of sexual differentiation	56	0.53
Duchenne muscular dystrophy	52	0.49
Palatal cleft	49	0.46
Achondroplasia	46	0.43
Unbalanced chromosome alteration	45	0.42
Congenital heart disease	41	0.39
Marfan syndrome	40	0.38
Congenital multiple arthrogryposis	39	0.37
Overgrowth syndrome	38	0.36
Mucopolysaccharidosis	36	0.34
Poland anomaly	35	0.33
Congenital cataract	34	0.32
Macrocephaly	34	0.32
Myopathy	33	0.31
Williams syndrome	32	0.3
Spinal muscular atrophy	31	0.29
Sequence of amniotic bands	31	0.29
Sensorineural hearing loss	30	0.28
Craniosynostosis	29	0.27
Syndromic lip‐palatal fissure	29	0.27
Cardiac malformation	28	0.26
Beckwith‐Wiedemann syndrome	24	0.23
Crouzon syndrome	24	0.23
Russell‐Silver syndrome	23	0.22
Syndromic congenital heart disease	21	0.2
Tuberous sclerosis	21	0.2
Sotos syndrome	21	0.2
Epileptic encephalopathy	20	0.19
Cornelia de Lange syndrome	20	0.19
Syndromic microphthalmia	19	0.18
McCune‐Albright syndrome	18	0.17
Scoliosis	17	0.16
Leukodystrophy	17	0.16
Sensory‐motor polyneuropathy	17	0.16
Multiple enchondromatosis	16	0.15
Holoprosencephaly	16	0.15
Hypophosphatemic rickets	16	0.15
Congenital hypothyroidism	15	0.14
Congenital lipodystrophy	15	0.14
Microphthalmia	15	0.14
Polydactyly	15	0.14
Kabuki syndrome	15	0.14
Renal tubular acidosis	14	0.13
VACTERL Association	14	0.13
Syndromic cleft palate	14	0.13
Syndromic cardiac malformation	14	0.13
Meromelia	14	0.13
Micropene	14	0.13
Klippel–Feil syndrome	14	0.13
Prader–Willi syndrome	14	0.13
Waardenburg syndrome	14	0.13
Blepharophimosis syndrome—inverted epicanthus—palpebral ptosis	13	0.12
Cardiofaciocutaneous syndrome	13	0.12
Marshall syndrome	13	0.12
Spondylocostal dysostosis	12	0.11
Multiple exostosis	12	0.11
Congenital hydrocephalus	12	0.11
Syndromic macrocephaly	12	0.11
Apert syndrome	12	0.11
Parry‐Romberg syndrome	12	0.11
Cleidocranial dysostosis	11	0.1
Hypospasses	11	0.1
Cerebral infantile paralysis	11	0.1
Albinism	10	0.09
Lip fissure	10	0.09
Gastroschisis	10	0.09
Pierre‐Robin sequence	10	0.09
Cayler syndrome	10	0.09
Holt‐Oram syndrome	10	0.09
Radio‐ulnar synostosis	10	0.09
Brachydactyly	9	0.08
Ectodermal dysplasia	9	0.08
Congenital adrenal hyperplasia	9	0.08
Hypomelanosis of Ito	9	0.08
Primary immunodeficiency	9	0.08
Anorectal malformation	9	0.08
3MC syndrome	9	0.08
Costello syndrome	9	0.08
Klinefelter syndrome	9	0.08
Klippel‐Trenaunay‐Weber syndrome	9	0.08
Dilated cardiomyopathy	8	0.08
Hypohidrotic ectodermal dysplasia	8	0.08
Schizencephaly	8	0.08
Syndromic hemihyperplasia	8	0.08
Rubinstein–Taybi syndrome	8	0.08
Stickler syndrome	8	0.08
Anemia Fanconi	7	0.07
Intrahepatic cholestasis	7	0.07
Charcot‐Marie‐Tooth disease	7	0.07
Gaucher disease	7	0.07
Neuromuscular disease	7	0.07
Heterotaxia	7	0.07
Primary congenital microcephaly	7	0.07
Osteopetrosis	7	0.07
Spastic paraparesis in the family	7	0.07
Bardet‐Biedl syndrome	7	0.07
Moebius syndrome‐Anomaly Poland	7	0.07
Pallister‐Killian syndrome	7	0.07
Prune‐belly syndrome	7	0.07
Tricorinophalangeal syndrome	7	0.07
Trisomy 18	7	0.07
Ataxia‐telangiectasia	6	0.06
Syndromic congenital cataract	6	0.06
Mandibulofacial dysostosis	6	0.06
Faunatosis pigmentosa	6	0.06
Syndromic anorectal malformation	6	0.06
Hand held	6	0.06
Escobar syndrome	6	0.06
Treacher‐Collins syndrome	6	0.06
Aniridia	5	0.05
Incontinence pigmenti	5	0.05
Cystic lymphangioma	5	0.05
Angelman syndrome	5	0.05
Coffin‐Lowry syndrome	5	0.05
FATCO syndrome	5	0.05
Androgen Insensitivity syndrome	5	0.05
Crystalline subluxation	5	0.05
Ataxia	4	0.04
Glucose‐6‐phosphate dehydrogenase deficiency	4	0.04
Congenital hypotrichosis	4	0.04
Syndromic infantile cerebral paralysis	4	0.04
Fetal alcohol syndrome	4	0.04
CHARGE syndrome	4	0.04
Ehlers‐Danlos syndrome VI	4	0.04
Hallermann‐Streiff syndrome	4	0.04
Loeys‐Dietz syndrome	4	0.04
Proteus syndrome	4	0.04

Note

Courtesy: Dr. Hugo Abarca‐Barriga.

a Table does not show 2,743 diagnostic cases for others genetic disorders of unknown etiology.

Chromosomal microarray analysis, is the first‐tier clinical diagnostic test for patients with intellectual disability, autism spectrum disorders and congenital anomalies (Miller et al., 2010). This technique was implemented and developed at INSN‐Breña, revealing etiology in more than 50% of patients with intellectual disability and development delay (Abarca‐Barriga, Chávez Pastor, Trubnykova, Vásquez, & Poterico, 2017), with a previous clinical genetic consultation. Furthermore, we found novel variants and demonstrated how loss of copy number variants (CNV) resulted in homozygous deletion in *MPLKIP *(OMIM #609188) and *SUGCT* (OMIM #609187) genes, which are causes of Trichothiodystrophy type 4 and glutaric aciduria type 3, respectively (Serna‐Infantes et al., 2018). Also, it allowed us to describe a patient with 18p−/18q+ syndrome, adding to the phenotype by describing the persistent microscopic hematuria and aortic pseudocoarctation with descending aorta walls thickening, suggesting Takayasu's arteritis (Poterico et al., 2017).

Collaboration with European geneticists allowed the report of novel CNVs of the Sonic hedgehog (SHH) limb enhancer ZPA regulatory sequence (ZRS) on chromosome 7q36 and its relationship with specific polysyndactyly syndromes. These microduplications correlated to Laurin–Sandrow syndrome and Haas‐type polysyndactyly, according to the ZRS region length (Lohan et al., 2014). Moreover, Peruvian geneticists of INSN described unique cases of pterygium‐digital keloid dysplasia (Abarca et al., 2014), H syndrome (Abarca Barriga, Trubnykova, Polar Córdoba, Ramos Diaz, & Aviles, 2016), van der Knaap syndrome (Hamilton et al., 2018), and Shawaf‐Traboulsi syndrome (Abarca Barriga et al., 2018). Last, international collaboration permitted reports of two common genetic syndromes—that is, Noonan (Kruszka et al., 2018) and Williams‐Beuren (Kruszka et al., 2018)—with clinical geneticists of the National Human Genome Research Institute of The National Institutes of Health; and with other geneticists of different sites in the globe, contributing to the worldwide clinical genetics area.

A political decision allowed the Peruvian population to have a novel institution for children's healthcare. This specialized institute is called Instituto Nacional de Salud del Niño San Borja (INSN‐SB) and is a national reference center for complex cases requiring surgical treatment or transplantation. The Genetics Service started in 2015, with a rapid development in clinical and laboratory activities.

The clinical genetics consultations at INSN‐SB are carried out by five clinical geneticists, estimating 400 patients attending every month approximately. The most frequent diagnosis is Down syndrome. Evaluation includes an increasing number of patients with diagnosis of cardiac anomalies (e.g., heterotaxy, dilated cardiomyopathy), neurological (e.g., epilepsy, intellectual disability, hearing loss) or behavioral disorders (e.g., autism).

Cytogenetic analyses have been implemented since the establishment of the Genetics Service at INSN‐SB, mostly for constitutional analyses. In addition, there are bone marrow studies of children with leukemia, and chromosome fragility test for diagnosis of Fanconi anemia—being the unique center doing this test in Peru. Since 2016, more than 500 blood and almost 200 bone marrow samples have been analyzed. On the other hand, more than 600 samples have been analyzed for detection of genetic alterations in patients with haematological neoplasms. Ongoing research projects with institutional investment involve cardiac anomalies and pharmacogenetics in children with leukemia.

INSN‐SB is working on administrative and logistics issues for obtaining equipment and human resources for the implementation of genetic panel test using next‐generation sequencing (NGS) in pediatric diseases, such as cardiac anomalies, bone marrow failure syndromes, among others.

### 2.4 Cancer genetics

Clinical genetics and counseling in oncology started in 2006 at the Instituto Nacional de Enfermedades Neoplásicas (National Institute of Neoplastic Diseases—INEN) as a research initiative with public funding. Since that time, genetic consultations have been increasing not only in numbers but also in clinical conditions with internal and external collaborations, as more non‐genetics physicians are aware of hereditary conditions. Moreover, the clinical training on genetics has INEN as the unique node for future medical geneticists.

More than 1,500 patients have attended a clinical geneticist consultation; even if reference to the genetic consultation at INEN is <5% annually. For instance, breast cancer represents the more frequent consultation at the Unit of Molecular Biology and Genetics at INEN (Manrique, Sullcahuamán‐Allende, & Limache‐García, 2013). Collaboration with foreign institutions such as the private Clinic City of Hope, lead the Peruvian clinical geneticists to be part of clinical research on hereditary or familial cancer in Peru.

Abugattas et al. (2015) depicted in an unselected cohort of women with breast cancer that 5% carried *BRCA1* (OMIM #113705) or *BRCA2* (OMIM #600185) specific genetic alterations (Latino‐variants panel). *BRCA1 185delAG* was the most frequent variant in these patients. However, the technology employed to identity genetic chances involved a catalog of Latino‐variants confirmed by Sanger sequencing, not involving other types of pathogenic genetic changes. Long‐standing collaboration with City of Hope has been allowing—through a continuous research project—reports of germline variants of patients attending the genetic counseling consultation at INEN. Genetic information helps geneticists to give an appropriate genetic counseling, and an appropriate management.

Current state‐of‐the art technologies (i.e., NGS) locally implemented at INEN would allow more patients for evaluation and genetic counseling. On the other hand, clinical and laboratory data have allowed Peruvian geneticists to establish international collaborations not only on the area of hereditary of familial breast cancer syndromes (Abugattas et al., 2015), but also on colorectal cancer (CRC) (Rossi et al., 2017) and gastric cancer (GC) predisposition (Slavin et al., 2017), among others.

Moreover, other clinical international collaborations have arisen on CRC and GC. For instance, a large study which included 362 GC patients from Lima, Peru fond that *IRF4 *(OMIM #601900), *ELMO1*(OMIM #606420), *CLIP4* (OMIM #605736) and *MSC* (OMIM #603628) promoter methylation coupled with a GDMI > 4 are useful molecular tools for GC risk stratification in endoscopic biopsies (Pirini et al., 2017). An interesting study in native Peruvians (Quechuas from the Central Andes) did not found an association between candidate SNPs and GC (Zamudio et al., 2016).

Twenty percent of the Latin American countries have developed guidelines for early detection of CRC, and in the last years with the advent of NGS there have been several tests performed to detect hereditary cancer syndromes, especially Lynch syndrome. These studies in Latin America have reported about 18% of patients diagnosed at a young age have pathogenic variants in genes not traditionally associated with CRC (e.g., *ATM *[OMIM #607585], *CHEK2 *[OMIM #604373], *BRCA1*, *BRCA2*, *CDKN2A *[OMIM #600160], *PALB2 *[OMIM #610355]). *MHL1* (OMIM #120436) pathogenic variants were more frequent in patients from Peru, Mexico and Chile, whereas *MSH2* (OMIM #609309) pathogenic variants occurred mostly in patients from Colombia and Argentina (Ñique Carbajal, Sánchez Renteria, Lettiero, Wernhoff, & Domínguez‐Valentin, 2014; Rossi et al., 2017).

### 2.5 Genetics in autoimmune diseases

Several manuscripts have been published on genetic aspects of autoimmune diseases in Peruvian populations; most of them were collaborative efforts. These studies involved patients of tertiary‐level hospitals, mainly the Hospitals Guillermo Almenara Irigoyen and Edgardo Rebagliati Martins from the Social Security System, and in the Hospital Cayetano Heredia from the Ministry of Health System. Specific studies relied on autoimmune disorders, such as rheumatoid arthritis (RA), systemic lupus erythematosus (SLE), spondyloarthritis (SpA) and autoimmune hepatitis.

A new variant *HLA‐DRB1* (OMIM #142851) (14:02) was reported in the first of this sort of Peruvian studies on RA in 2001 (Castro et al., 2001). A meta‐analysis of *HLA‐DQA1* (OMIM #146880) polymorphism in Latin American populations included two Peruvian reports on this issue: from the Hospital Guillermo Almenara Irigoyen and the Air Force Army Hospital (Delgado‐Vega & Anaya, 2007). Another Latin American collaboration—Genome of RA network consortium (GENAR)—found that the more Amerindian ancestry, the lower odds of fulfilling several RA criteria (Sánchez et al., 2017). On the other hand, Amerindian descendants could have new genetic loci related to RA (Herráez et al., 2013).

In SLE, two Peruvian hospitals (Hospitals Guillermo Almenara Irigoyen and Edgardo Rebagliati Martins) are part of GENLES (Genoma de Lupus Eritematoso Sistémico Network consortium/Genome of SLE network consortium) which is the biggest genetic study in Latin American populations. This cohort has reported that Amerindian ancestry relates to higher genetic load of SLE risk alleles compared to Caucasian (Sanchez et al., 2010) and to a more severe disease (Sánchez et al., 2012). Nevertheless, when two cohorts with a similar ancestry were compared (LUMINA [Lupus in Minorities: Nature vs. Nurture] and GLADEL [Grupo Latino Americano de Estudio de Lupus/Latin American Group for the Study of SLE]), outcome differences could not be explained by Amerindian ancestry (Ugarte‐Gil et al., 2015). Furthermore, GENLES, in collaboration with other cohorts from US and Europe, reported several new loci (Langefeld et al., 2017; Ugarte‐Gil et al., 2015). As part of a Peruvian SLE Cohort named the Almenara Lupus Cohort, a study of hypomethylation in SLE patients has been started in collaboration with the University of California in San Francisco (UCSF).

Regarding SpA and its main genetic risk (i.e., *HLA‐B27 *[OMIM #142830]), a Peruvian population from Hospital Cayetano Heredia in 1981 (Alarcon et al., 1981) reported a low frequency of *HLA‐B27* antigen. This asseveration affects SpA Peruvian patients (Cuchacovich et al., 2002), explaining in part the low proportion of Peruvian patients suffering with SpA with this genetic marker. Recently, Hospital Guillermo Almenara Irigoyen has found that ankylosing spondylitis patients in Peru not only have a lower prevalence of *HLA‐B27* (<30%) but also a high prevalence of *HLA‐B39* (almost 50%); the latter variant is expected to have a similar effect than *HLA‐B27* in the pathogenesis of the disease (F. Zevallos, MF. Ugarte‐Gil, RV. Gamboa‐Cardenas, M. Medina‐Chinchon, JM. Cucho‐Venegas, JL. Alfaro‐Lozano, Z. Rodriguez‐Bellido, C. Pastor‐Asurza, R. Perich‐Campos, unpublished data).

In autoimmune hepatitis, a cohort from the Hospital Guillermo Almenara Irigoyen showed *HLA‐DR4* (OMIM #600185) and *HLA‐DR13* markers as the most frequent, similarly to other Latin American countries. Conversely, European‐descendant studies reported *HLA‐DR3* and *HLA‐DR4 *as the most frequent (Cárdenas Ramírez et al., 2018; Padilla et al., 2012).

Furthermore, Peruvian patients—regarding other autoimmune diseases—from the Hospital Edgardo Rebagliati Martins have been also been included in several multinational reports of primary immunodeficiencies (Stray‐Pedersen et al., 2017), including *STAT1 *(OMIM #600555) mutations (Aldave Becerra & Cachay, 2017; Toubiana et al., 2016), hemophagocytic lymphohistiocytosis (Chinn et al., 2018), Wiskott–Aldrich syndrome and X‐linked thrombocytopenia (Crestani et al., 2015).

### 2.6 Neurogenetics research

Neurogenetics in Peru offers specialized care for patients and families affected with repeat expansion disorders and other familial neurodegenerative disorders. The only neurogenetics center in Peru, based at the Instituto Nacional de Ciencias Neurologicas (National Institute of Neurological Sciences), was founded in 1995. The Neurogenetics center provides specialized care for patients and families affected with genetic and orphan neurodegenerative disorders (Cornejo‐Olivas, Espinoza‐Huertas, et al., 2015). By 2000, the CAG repeat analysis of *HTT *(OMIM #613004)*,* the gene responsible for Huntington disease became the first molecular protocol implemented at this center (Walker et al., 2018). As a national reference center, the outpatient clinic receives patients and families from different regions of Peru, mostly affected with Huntington disease, inherited ataxias, myotonic dystrophy type 1 and other muscular dystrophies, inherited neuropathies, familial Parkinson and Alzheimer disease among others (Cornejo‐Olivas, Espinoza‐Huertas, et al., 2015; Mazzetti et al., 2015; Walker et al., 2018). Since 2009, neurogenetic care combines multidisciplinary clinical approach, molecular diagnosis, and clinical research.

Large samples of Huntington and inherited ataxia cases provide a better understanding of genetic epidemiology and its impact in public health in these disorders. The first report of Huntington disease, also known as CHOR‐HTT, in Peru traces back to 1950, describing a large family from southern Peru (Torres, Mori‐Quispe, & Mendoza‐Cabanillas, 2006). Further reports during the late 80s and early 90s highlighted a surprisingly high frequency of cases coming from the Cañete Valley—southern Lima, 200 km south to the Capital city (Cuba, 1986; Cuba, Castro, & Benzaquen, 1983; Cuba & Torres, 1989). The large cohort of CHOR‐HTT families in Peru have been made significant scientific contributions in the field including genotype‐phenotype correlations of late‐onset cases (Cornejo‐Olivas, Inca‐Martinez, et al., 2015) and Huntington‐like disorders (Walker et al., 2018), haplotypes analysis suggesting an Amerindian origin of the HTT mutation (Kay et al., 2017) and health‐cost analysis highlighting the dramatic burden of CHOR‐HTT in the Peruvian population. Since 2012, an outreach clinic, combining both home visits and consultations at primary care center, is offering multidisciplinary care to CHOR‐HTT patients and families from the Cañete valley (Vishnevetsky et al., 2018). Inherited ataxic cases followed over the past 10 years is demonstrating a different distribution of dominant ataxias with relative high frequency of spinocerebellar ataxia type 10 or SCA10 (OMIM #603516) in Peruvian population (Bampi et al., 2017; Gheno et al., 2017).

Genetics of Parkinson disease (PD) demonstrates the transcendence of studying populations with different ancestries when looking for pathogenic variants. Since 2007, as part of the Latin American Research Consortium on the Genetics of Parkinson's Disease (LARGE‐PD), the study of genetics of PD in Peruvian population contributed to increased knowledge of PD. The LARGE PD cohort in Peru facilitate the discovery of novel mutations in *PARKIN* (OMIM #602544) gene (Cornejo‐Olivas, Torres, et al., 2015) as well as addressing the frequency and genotype‐genotype correlations of pathogenic variants of the *LRRK2 *(OMIM #609007) gene in Mestizo population from Latin America (Cornejo‐Olivas et al., 2017; Mata et al., 2009, 2011).

### 2.7 Genetics and pharmacogenetics in tuberculosis

Tuberculosis (TB) is an infectious disease with high incidence in Peru. It has been shown that TB can occur at different rates among different races, ethnicities, and even families indicating a genetic predisposition to tuberculosis susceptibility. Different studies have been done to identify genetic polymorphisms related to severity and progression of TB disease. Two‐locus genotype −2,518 macrophage chemoattractant protein *CCL2 *(OMIM #158105) GG and −1607 matrix metalloproteinase *MMP1*(OMIM #120353) 2G/2G has been found to promote the expression of hyperinflammation in response to *Mycobacterium tuberc*ulosis infection, inducing extensive tissue damage and a severe TB disease (Ganachari, Guio, Zhao, & Flores‐Villanueva, 2012). Regarding TB progression, some variants located near the enhancer region on chromosome 3q23 also have been found (Luo et al., 2018). Together with the data obtained from the Peruvian Genome Project and these previous studies, it will be possible to define the origins and distribution of these new polymorphisms associated with transmissible and non‐transmissible diseases in Peru: integrating Population Genomics knowledge to Public Health.

How our genetic diversity affects our pharmacological therapy is the basis of pharmacogenomics where the genotype of an individual is identified to determine his or her drug metabolic phenotype, predicting the probability of his or her response and minimizing the adverse reactions to achieve a personalized treatment (Rohrer Vitek, Nicholson, Schultz, & Caraballo, 2015; Weinshilboum & Wang, 2004). Even though metabolic genotypes and phenotypes have been widely studied in Caucasian and Asian populations, in many Latin American populations this key information is still missing. The Peruvian Mestizo population's unique admixture between multiple Native American communities in its region (Harris et al., 2018) results in a distinct population or populations that do not allow for assumptions from existing pharmacogenomics data. Up to date, only two pharmacogenomic studies have included Peruvian Native American samples (Bisso‐Machado et al., 2016; Fuselli et al., 2007). Due to Peru's high rate of TB, researchers are directing their focus toward the role of pharmacogenomics on TB treatment by starting with the identification of the genetic polymorphisms affecting isoniazid and rifampicin (first‐line and second‐line anti‐tuberculous drugs) metabolism and thus affecting the efficacy of the treatment and the induction of adverse effects. Two main research groups (Dr. Heinner Guio's group at the Peruvian National Institute of Health in collaboration with the Peruvian National Health Strategy for the Prevention and Control of Tuberculosis [ESPNCT[ Guio, Levano, Sánchez, & Tarazona, 2015], and Dr. Ricardo Fujita's group at the San Martin de Porres University) are identifying the frequency in the Peruvian population of genetic polymorphisms in crucial drug metabolizing enzymes, *NAT2* (OMIM #612182) and *CYP2E1* (OMIM #124040), that result in phenotypes that could cause either a decrease or an increase in anti‐tuberculous drug concentration affecting the efficacy of the treatment. Genotypic and phenotypic assessment will become essential for the evaluation protocol to provide an adequate personalized treatment that increases efficacy and reduces side effects. The concept of “one dose for all” strategy in TB treatment could be an alternative to avoid mono or multidrug resistance in the *Mycobacteria tuberculosis.*

## 3 BASIC AND TRANSLATIONAL RESEARCH ON GENETICS AND GENOMICS

### 3.1 An example of the role of Peruvian universities in genetic and genomic research

The Centro de Genetica y Biologia Molecular de la Universidad San Martin de Porres (CGBM‐USMP) has conducted studies on rare Mendelian diseases, chronic diseases, pharmacogenetics, immunogenetics and ancestry in Peruvian populations. As expected, in a mestizo population, there are germline variants reported in Europe, Africa, and Asia, whereas other novel variants, probably of Amerindian origin are also frequently found. Some Mendelian diseases studied by this university‐research center are ocular, neuromuscular diseases, cancer, among others.

CGBM‐USMP studies of glaucoma in different Peruvian families have allowed refinement of the *GLC1B* (OMIM #606689) locus in the 2q12‐q13 chromosomal region, and a candidate gene for the disease is currently being assessed. The study of *MYOC *(OMIM #601652) (locus *GLC1A*) has identified both known mutations in European and African‐American populations, as well as novel mutations. Interestingly, these variants are correlated with African ancestry (Chincha and Cañete) and the novel are recurrent in Peru, probably indicating an autochthonous founder effect (Guevara‐Fujita et al., 2008; Mendoza‐Reinoso et al., 2012). Another disease studied at CGBM‐USMP is retinitis pigmentosa—which has a genetic heterogeneity, with more than 70 genes—in approximately 100 Peruvian families, having identified the causative mutation in 30 of them. Notably, the Parán community—enclave in a rural area of Lima—with 60 of the 350 inhabitants have retinitis pigmentosa due to germline variants in the *RPGR* (OMIM #312610) gene located on the chromosome X.

CGBM‐USMP team has assessed more than 300 Peruvian patients with muscular dystrophies, most of them with clinical diagnosis of Duchenne disease. The most common type of mutation are exon deletions, although much lower than in other studies (38% vs. 55%–70%). On the other hand, nonsense mutations have a higher prevalence (30%) compared to other reports (10%–15%). It is not known the reason of these results, but it is possible that it is due to founder effects. This group's research on Peruvian patients with breast cancer has been depicting interesting results. A first analysis of *BRCA1* and *BRCA2* by Sanger sequencing and multiplex ligation‐dependent probe amplification (MLPA) in 18 families with suspicion of hereditary breast and ovary cancer syndrome (HBOC), demonstrated four pathogenic variants. Three of these variants have been previously reported (*BRCA1*: c.302–1G>C and c.815_824dup10; *BRCA2*: c.5946delT), but they are absent in the Latino‐variants panel mentioned above. On the other hand, a duplication of adenines in exon 15 in *BRCA1 *(c.4647_4648dupAA, ClinVar SCV000256598.1) and *BRCA1* c.4647_4648dupAA were novel pathogenic variants. Interestingly, two exonic and four intronic variants of unknown significance, some of them found recurrently in HBOC Peruvian families and are absent in normal controls and databases (Buleje et al., 2017). Additionally, other universities such as the Universidad Peruana Cayetano Heredia and the Universidad Mayor de San Marcos have made important contributions in genetic and genomic research.

### 3.2 Fundamentals of biobanking for translational oncology research

Instituto Nacional de Enfermedades Neoplásicas is a public health center, specialized in the prevention, detection, diagnosis, treatment, and rehabilitation of patients affected by neoplastic diseases or malignant tumors. INEN have implemented genetic platforms for diagnosis and research with international collaborations. The administrative area directly linked to development of oncogenomic scientific research in the institute is the Tumor Biobank of INEN (BTT).

BTT collaborates with diverse scientific organizations, hence serving as a platform for translational research in oncology, with implemented laboratories with capacities in: molecular biology, cell culture, image registration, and histotechnology.

In collaboration with the Institute of Research for Development (IRD)—France, BTT researchers described in Peru an unusual clinical presentation of hepatocellular carcinoma (HCC) with noteworthy characteristics compared to those observed in other regions of the world. We described a younger population displaying an abnormal juvenile mean age of 25 years old, and large tumor exceeding 10 cm diameter, and only 11% of HCCs occurred in the setting of cirrhosis (Bertani et al., 2013; Ruiz et al., 2016).

In order to deepen our understanding of the molecular processes ongoing in Peruvian liver tumors, mutation spectrum analysis was carried out on hepatocellular carcinomas from Peruvian patients. Thirty‐one exons covering 17 kb in nine genes considered as mutation hotspots in HCC were analyzed: *ARID2 *(OMIM #609539)*, AXIN1*(OMIM #603816)*, BRAF *(OMIM #164757)*, CTNNB1 *(OMIM #116806)*, NFE2L2 *(OMIM #600492)*, H‐RAS (*OMIM #190020) *K‐RAS *(OMIM #190070), *N‐RAS *(OMIM #164790), and *TP53 *(OMIM #191170) (Marchio et al., 2014). Such genotypic alterations are unusual elsewhere, as *CTNNB1* mutants were found preferentially in non‐HBV‐associated HCC. The uncommon genetic profile of Peruvian HCC was further confirmed by the conspicuous excess of deletions present in the mutation spectrum, a pattern rarely observed in sporadic cancer and never described so far in HCC (Marchio et al., 2014). The bimodal age‐based distribution, the young age of a distinct subgroup, the low rate of associated cirrhosis, and a peculiar mutation spectrum delineate an unusual tumoral process ongoing in the Peruvian population.

In the last years, the pediatric department of INEN received an unusually high number of children with primary intracranial sarcomas. Most of them are fusocellular and pleomorphic high‐grade sarcomas. BTT with international collaboration allow analysis with NGS of 11 pediatric intracranial sarcomas NOS from Peru; revealing DICER1 (OMIM #606241) pathogenic variants in terms of a distinct methylation signature and the presence of DICER1 alterations (Koelsche et al., 2018).

### 3.3 Understanding molecular behavior for genetics

Genetic and genomic medicine need the feedback—and vice versa—from basic science for understanding molecular, cellular, and intercellular processes and applications to clinical settings (Shamanna, Croteau, Lee, & Bohr, 2017). Werner protein (WRNp) represents an excellent example of this, with a scientific node in Peru with international collaboration. Werner syndrome (WS) is a rare autosomal recessive and heritable disease with unknown prevalence in Latin American countries, among others. This disease is associated with truncation or non‐functional variants of the protein (Orphanet, 2012). Therefore, patients with this condition are prone to premature aging: that is, metabolic disturbances, cardiovascular defects and neoplastic diseases (Orphanet, 2012; Shamanna et al., 2017). Among the latter, a higher risk of a small subset of neoplasms has been reported, with several specific types of cancer reported like thyroid cancer, malignant melanoma, meningioma, soft tissue sarcoma, osteosarcoma, breast cancer, and leukemia (Goto & Ishikawa, 1996; Lauper, Krause, Vaughan, & Monnat, 2013).

It is well known that WRNp is a nuclear protein whose main function is to protect genome integrity (Rossi, Ghosh, & Bohr, 2010), but it has also been detected in the cytoplasm (Kobbe et al., 2002; Shamanna et al., 2016; Slupianek et al., 2011). However, the cytoplasmatic WRNp behavior remains unknown. Tokita et al. recently reported in a genomic evaluation that tumors of patients with WS depicted slight differences in senescence, mitochondrial dysfunction and telomere attrition markers (Tokita et al., 2016). Interesting but uncertain—according to specific population findings—clinical prediction related to WRN dysregulation in some diseases such as cancer, remains to be clarified with WRNp variants different from the truncating or non‐functional. For instance, reports have suggested a link between this sort of variants and breast cancer (Wang et al., 2009; Wirtenberger et al., 2006). Indeed, more research of WRNp needs to be carried out to clarify clinical consequences and therapeutic targets development.

Peruvian Professor Iglesias‐Pedraz and his foreign colleagues have been trying to elucidate the WRNp function and behavior, demonstrating that immortal cancer cells harboring stable and conditional silencing system for WRNp, reduce de novo protein synthesis prior to any detectable stress response, weakening the cellular proliferation. This alteration is not due to a defect in gene expression nor protein instability (Li et al., 2014), but due apparently to a defect in the proper protein synthesis in WRN‐depleted cancer cells.. Additional findings have been observed by these scientists; for example, they found WRNp located in the cytoplasm co‐sediments with ribosomal‐enriched complex (unpublished data). This finding was noticed years before by Motonaga et al. (2002) in human tissues and recently, Lachapelle et al. (2011) have identified interaction of WRNp with several components of translational machinery. The aforementioned suggests that the collaborating‐group of Professor Iglesias‐Pedraz could be immersed in an exciting and novel scientific road regarding WRNp.

## 4 PERUVIAN GENOME PROJECT

Modern Peruvians carry in their DNA the history of 2.5 million years of evolution and 150,000 years of migration from Africa to Peru or the Americas. Climatic changes and high altitude, different pathogens and interaction with other ethnic groups, have sculpted the genetic variants and genetic expression in the modern Peruvians. The Andes mountains divide Peru in three regions (coast, highlands, and jungle). One third of the Peruvian population lives above 2,500 m.a.s.l. and over 50,000 people reside above 4,000 m.a.s.l (Macsorley, 2001). On the other hand, 60% of the Peruvians that live in the jungle are exposed to different intestinal parasites leaving a particular human being homeostatic condition. Thus, Peruvians represent an unexplored genomic book with unknown genome‐environment interaction data.

By 2011, the Peruvian National Institute of Health (Instituto Nacional de Salud del Peru) started the Peruvian Genome Project lead by Dr. Heinner Guio in collaboration with Timothy O'Connor from University of Maryland and Dr. Tarazona from Universidade Federal de Minas Gerais. Despite disparities in research cost (e.g., equipment and supplies) as compared to US (more than 2.5 times) (Sirisena & Dissanayake, 2018), a total of 280 genomes from 17 Native and 13 Mestizo populations have been analyzed (150 genomes were sequenced using whole genome sequencing and 130 using genotype array platform). This study represents the most extensive Native American sequencing project to date. The analysis shows that our Mestizo populations have 60%–70% native genes—in some geographical locations more than 80%. Most of the newly identified SNPs are missense (~60,000) and come from native communities (Harris et al., 2018). Our data demonstrate a high Native ancestry component in the Peruvian population, even as compared to the results obtain in the Mexican genomic project (Belbin, Nieves‐Colón, Kenny, Moreno‐Estrada, & Gignoux, 2018). Additional studies in genomics of Peruvian populations will help us to understand these new SNPs (Elhaik et al., 2014; Sandoval et al., 2013). No doubt, these new findings from the Peruvian Genome project will enhance genetic research and medical genomics not only for Peruvians, but for Hispanics and Latinos.

## 5 CONCLUDING REMARKS

Peru has been working on genetics and genomics despite the lack of national investment. Multidisciplinary healthcare providers have been contributing to the area of genetics in Peru. We have highlighted several examples of achievements in the clinical and research arenas. Moreover, as the number of well‐trained professionals increases—*in house* and abroad—contributing with national and international collaborators, research, and clinical experiences will increase in quality and extension. All the research data shown in this review could not have been done without local and/or international collaborations. In these times where lots of standardized and incoming technologies and data are emerging, collaboration is extremely important for research and its translation to the public health system, helping in the implementation of precision medicine.

## CONFLICT OF INTEREST

None declared.
